# 2-Hydr­oxy-*N*′-(2-methoxy­naphthyl­idene)benzohydrazide

**DOI:** 10.1107/S1600536808026962

**Published:** 2008-08-23

**Authors:** Xiao-Yang Qiu

**Affiliations:** a Department of Chemistry, Shangqiu Normal University, Shangqiu 476000, People’s Republic of China

## Abstract

The title Schiff base compound, C_19_H_16_N_2_O_3_, prepared by the reaction of 2-meth­oxy-1-naphthyl­aldehyde and 2-hydroxy­benzohydrazide, crystallizes with two independent mol­ecules in the asymmetric unit. Each mol­ecule exists in a *trans* configuration with respect to the methyl­idene group. The naphthyl ring system make dihedral angles of 65.0 (2)° and 55.8 (2)° with the planes of the benzene rings. Intra­molecular N—H⋯O and O—H⋯O hydrogen bonds help to stabilize the mol­ecular conformations. In the crystal structure, mol­ecules are linked into one-dimensional chains parallel to the *c* axis by inter­molecular O—H⋯N and O—H⋯O hydrogen bonds.

## Related literature

For the biological properties of hydrazones, see: Bedia *et al.* (2006 ▶); Rollas *et al.* (2002 ▶); Fun *et al.* (2008 ▶). For our previous reports on hydrazones, see: Qiu, Fang *et al.* (2006 ▶); Qiu, Luo *et al.* (2006*a* ▶,*b* ▶); Qiu, Xu *et al.* (2006 ▶). For bond-length data, see: Allen *et al.* (1987 ▶). For related structures, see: Singh *et al.* (2007 ▶); Narayana *et al.* (2007 ▶); Cui *et al.* (2007 ▶); Diao *et al.* (2008 ▶).
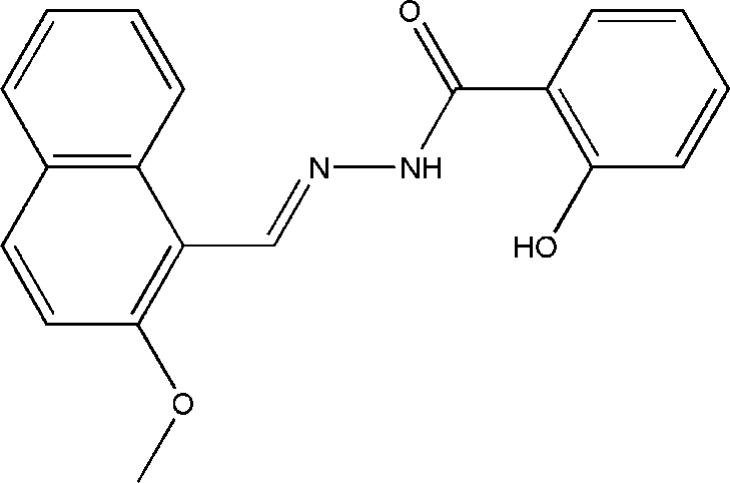


## Experimental

### 

#### Crystal data

C_19_H_16_N_2_O_3_
*M* *_r_* = 320.34
Monoclinic, 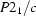

*a* = 15.186 (3) Å
*b* = 9.059 (2) Å
*c* = 24.135 (3) Å
β = 108.331 (3)°
*V* = 3151.8 (10) Å^3^
*Z* = 8
Mo *K*α radiation
μ = 0.09 mm^−1^
*T* = 298 (2) K
0.10 × 0.08 × 0.07 mm

#### Data collection

Bruker SMART CCD diffractometer
Absorption correction: multi-scan (*SADABS*; Sheldrick, 1996 ▶) *T*_min_ = 0.991, *T*_max_ = 0.994
17807 measured reflections
6805 independent reflections
4792 reflections with *I* > 2σ(*I*)
*R*_int_ = 0.021

#### Refinement

*R*[*F* ^2^ > 2σ(*F* ^2^)] = 0.043
*wR*(*F* ^2^) = 0.120
*S* = 1.03
6805 reflections
443 parameters
2 restraints
H atoms treated by a mixture of independent and constrained refinement
Δρ_max_ = 0.20 e Å^−3^
Δρ_min_ = −0.21 e Å^−3^

### 

Data collection: *SMART* (Bruker, 2001 ▶); cell refinement: *SAINT* (Bruker, 2001 ▶); data reduction: *SAINT*; program(s) used to solve structure: *SHELXS97* (Sheldrick, 2008 ▶); program(s) used to refine structure: *SHELXL97* (Sheldrick, 2008 ▶); molecular graphics: *SHELXTL* (Sheldrick, 2008 ▶); software used to prepare material for publication: *SHELXTL*.

## Supplementary Material

Crystal structure: contains datablocks I, global. DOI: 10.1107/S1600536808026962/at2612sup1.cif

Structure factors: contains datablocks I. DOI: 10.1107/S1600536808026962/at2612Isup2.hkl

Additional supplementary materials:  crystallographic information; 3D view; checkCIF report

## Figures and Tables

**Table 1:** Hydrogen-bond geometry (Å, °)

*D*—H⋯*A*	*D*—H	H⋯*A*	*D*⋯*A*	*D*—H⋯*A*
O3—H3⋯N3^i^	0.82	2.24	3.0049 (18)	155
O3—H3⋯O5^i^	0.82	2.30	2.914 (2)	133
O6—H6⋯O2	0.82	1.87	2.6683 (17)	164
N2—H2⋯O3	0.918 (19)	1.942 (16)	2.6518 (18)	132.7 (17)
N4—H4*A*⋯O6	0.895 (9)	1.948 (16)	2.6581 (19)	135.0 (18)
N4—H4*A*⋯O4	0.895 (9)	2.38 (2)	2.877 (2)	114.9 (16)

Symmetry code: (i) 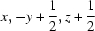.

**Fig. 1.**
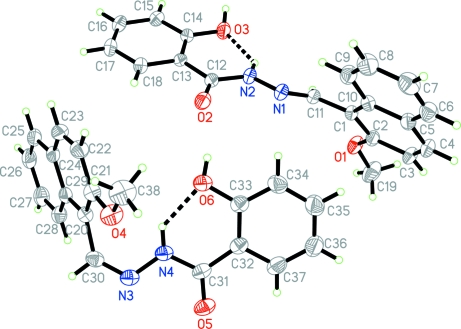
The molecular structure of (I), with atom labels and 30% probability displacement ellipsoids for non-H atoms.

**Fig. 2.**
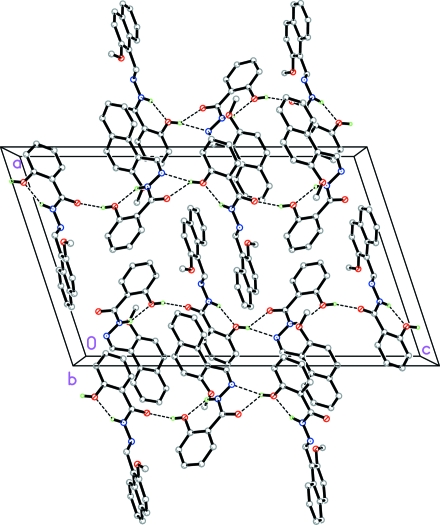
The crystal packing and hydrogen bonding of (I), viewed along the b axis.

## supplementary crystallographic information

### Comment

Hyrazone compounds, which derived from the reaction of aldehydes with hydrazides, have been widely studied due to their excellent biological properties (Bedia *et al.*, 2006; Rollas *et al.*, 2002; Fun *et al.*, 2008). Recently, we have reported a few Schiff hydrazone compounds (Qiu, Fang *et al.*, 2006; Qiu, Luo *et al.*, 2006*a*,b; Qiu, Xu *et al.*, 2006), we report herein the crystal structure of the title new compound, (I).

Compound (I) crystallizes with two independent molecules in the asymmetric unit (Fig. 1). Each molecule exists in a *trans* configuration with respect to the methylidene group. The naphthyl rings make dihedral angles of 65.0 (2)° and 55.8 (2)° with the planes of the benzene rings. The bond lengths and angles in (I) are found to have normal values (Allen *et al.*, 1987) and comparable to the values in the similar compounds (Singh *et al.*, 2007; Narayana *et al.*, 2007; Cui *et al.*, 2007; Diao *et al.*, 2008). Intramolecular O–H···O and N–H···O hydrogen bonds (Table 1) help to stabilize the molecular conformations. In the crystal structure, molecules are linked into one-dimensional chains parallel to the *c* axis by intermolecular O–H···N and O–H···O hydrogen bonds (Table 1 and Fig. 2).

### Experimental

The title compound was prepared by the Schiff base condensation of equimolar (0.5 mmol each) 2-methoxy-1-naphthylaldehyde and 2-hydroxybenzohydrazide in methanol (20 ml). Excess methanol was removed from the reaction mixture with distillation. The colourless solid was filtered and dried in air. Colourless block-shaped crystals suitable for X-ray diffraction were obtained from a methanol solution.

### Refinement

The imino H atoms were located in a difference map and refined with N–H distances restrained to 0.90 (1) Å. The remaining H atoms were positioned geometrically [C–H = 0.93–0.96 Å, O–H = 0.82 Å] and refined using a riding model, with *U*_iso_(H) = 1.2*U*_eq_(C) and 1.5*U*_eq_(C_methyl_ and O_hydroxyl_). Rotating group models were used for the methyl groups.

### Crystal data

**Table:**

C_19_H_16_N_2_O_3_	*F*_000_ = 1344
*M**_r_* = 320.34	*D*_x_ = 1.350 Mg m^−^^3^
Monoclinic, *P*2_1_/*c*	Mo *K*α radiation λ = 0.71073 Å
Hall symbol: -P 2ybc	Cell parameters from 5815 reflections
*a* = 15.186 (3) Å	θ = 2.4–28.1º
*b* = 9.059 (2) Å	µ = 0.09 mm^−^^1^
*c* = 24.135 (3) Å	*T* = 298 (2) K
β = 108.331 (3)º	Block, colourless
*V* = 3151.8 (10) Å^3^	0.10 × 0.08 × 0.07 mm
*Z* = 8	

### Data collection

**Table:**

Bruker SMART CCD diffractometer	6805 independent reflections
Radiation source: fine-focus sealed tube	4792 reflections with *I* > 2σ(*I*)
Monochromator: graphite	*R*_int_ = 0.021
*T* = 298(2) K	θ_max_ = 27.0º
ω scans	θ_min_ = 1.8º
Absorption correction: multi-scan(SADABS; Sheldrick, 1996)	*h* = −10→19
*T*_min_ = 0.991, *T*_max_ = 0.994	*k* = −11→11
17807 measured reflections	*l* = −30→30

### Refinement

**Table:**

Refinement on *F*^2^	Secondary atom site location: difference Fourier map
Least-squares matrix: full	Hydrogen site location: inferred from neighbouring sites
*R*[*F*^2^ > 2σ(*F*^2^)] = 0.043	H atoms treated by a mixture of independent and constrained refinement
*wR*(*F*^2^) = 0.120	*w* = 1/[σ^2^(*F*_o_^2^) + (0.0503*P*)^2^ + 0.6715*P*] where *P* = (*F*_o_^2^ + 2*F*_c_^2^)/3
*S* = 1.03	(Δ/σ)_max_ < 0.001
6805 reflections	Δρ_max_ = 0.20 e Å^−^^3^
443 parameters	Δρ_min_ = −0.21 e Å^−^^3^
2 restraints	Extinction correction: none
Primary atom site location: structure-invariant direct methods	

### Special details

**Table:**

Geometry. All e.s.d.'s (except the e.s.d. in the dihedral angle between two l.s. planes) are estimated using the full covariance matrix. The cell e.s.d.'s are taken into account individually in the estimation of e.s.d.'s in distances, angles and torsion angles; correlations between e.s.d.'s in cell parameters are only used when they are defined by crystal symmetry. An approximate (isotropic) treatment of cell e.s.d.'s is used for estimating e.s.d.'s involving l.s. planes.
Refinement. Refinement of *F*^2^ against ALL reflections. The weighted *R*-factor *wR* and goodness of fit *S* are based on *F*^2^, conventional *R*-factors *R* are based on *F*, with *F* set to zero for negative *F*^2^. The threshold expression of *F*^2^ > σ(*F*^2^) is used only for calculating *R*-factors(gt) *etc*. and is not relevant to the choice of reflections for refinement. *R*-factors based on *F*^2^ are statistically about twice as large as those based on *F*, and *R*- factors based on ALL data will be even larger.

### Fractional atomic coordinates and isotropic or equivalent isotropic displacement parameters (Å^2^)

**Table:**

	*x*	*y*	*z*	*U*_iso_*/*U*_eq_	
O1	0.43570 (8)	0.65866 (12)	0.40673 (6)	0.0577 (3)	
O2	0.24611 (8)	0.06121 (13)	0.35547 (5)	0.0573 (3)	
O3	0.15519 (9)	0.28893 (15)	0.47890 (6)	0.0654 (4)	
H3	0.1503	0.2849	0.5117	0.098*	
O4	0.16698 (9)	−0.14482 (14)	0.15763 (6)	0.0652 (4)	
O5	0.23207 (9)	0.34100 (14)	0.09458 (6)	0.0665 (4)	
O6	0.26324 (9)	0.11903 (14)	0.25093 (5)	0.0636 (3)	
H6	0.2693	0.1034	0.2854	0.095*	
N1	0.36544 (9)	0.26155 (15)	0.41827 (6)	0.0483 (3)	
N2	0.27814 (9)	0.24269 (15)	0.42336 (6)	0.0466 (3)	
N3	0.10594 (10)	0.13486 (16)	0.08670 (6)	0.0521 (4)	
N4	0.17195 (9)	0.16834 (16)	0.13899 (6)	0.0490 (3)	
C1	0.49640 (10)	0.42352 (17)	0.43517 (7)	0.0413 (4)	
C2	0.51078 (11)	0.56729 (17)	0.41994 (7)	0.0442 (4)	
C3	0.59831 (12)	0.61341 (19)	0.41810 (8)	0.0530 (4)	
H3A	0.6072	0.7102	0.4082	0.064*	
C4	0.66957 (12)	0.5156 (2)	0.43086 (8)	0.0556 (5)	
H4	0.7270	0.5469	0.4291	0.067*	
C5	0.65964 (11)	0.36863 (19)	0.44667 (7)	0.0483 (4)	
C6	0.73541 (13)	0.2690 (2)	0.46050 (8)	0.0613 (5)	
H6A	0.7926	0.3010	0.4586	0.074*	
C7	0.72615 (15)	0.1284 (2)	0.47645 (9)	0.0696 (5)	
H7	0.7763	0.0640	0.4854	0.084*	
C8	0.64025 (14)	0.0810 (2)	0.47939 (9)	0.0699 (6)	
H8	0.6341	−0.0155	0.4908	0.084*	
C9	0.56529 (13)	0.1723 (2)	0.46597 (8)	0.0576 (5)	
H9	0.5090	0.1366	0.4680	0.069*	
C10	0.57139 (11)	0.32084 (18)	0.44889 (7)	0.0439 (4)	
C11	0.40275 (10)	0.38697 (18)	0.43631 (7)	0.0434 (4)	
H11	0.3698	0.4561	0.4504	0.052*	
C12	0.22305 (11)	0.13587 (17)	0.39171 (7)	0.0436 (4)	
C13	0.13277 (10)	0.11120 (17)	0.40235 (7)	0.0421 (4)	
C14	0.10236 (11)	0.18085 (18)	0.44498 (7)	0.0470 (4)	
C15	0.01809 (12)	0.1424 (2)	0.45176 (8)	0.0574 (5)	
H15	−0.0014	0.1890	0.4802	0.069*	
C16	−0.03670 (13)	0.0369 (2)	0.41726 (9)	0.0644 (5)	
H16	−0.0929	0.0114	0.4225	0.077*	
C17	−0.00877 (13)	−0.0316 (2)	0.37477 (9)	0.0654 (5)	
H17	−0.0464	−0.1026	0.3509	0.078*	
C18	0.07502 (12)	0.00486 (19)	0.36748 (8)	0.0535 (4)	
H18	0.0934	−0.0425	0.3387	0.064*	
C19	0.44208 (15)	0.8020 (2)	0.38459 (12)	0.0814 (7)	
H19A	0.4892	0.8573	0.4129	0.122*	
H19B	0.3835	0.8515	0.3767	0.122*	
H19C	0.4579	0.7942	0.3492	0.122*	
C20	0.02531 (11)	−0.02550 (18)	0.13957 (7)	0.0456 (4)	
C21	0.08806 (12)	−0.12555 (18)	0.17276 (8)	0.0496 (4)	
C22	0.06931 (13)	−0.2047 (2)	0.21794 (8)	0.0573 (5)	
H22	0.1128	−0.2708	0.2405	0.069*	
C23	−0.01255 (13)	−0.1841 (2)	0.22840 (8)	0.0568 (5)	
H23	−0.0247	−0.2383	0.2579	0.068*	
C24	−0.07948 (11)	−0.08342 (18)	0.19611 (7)	0.0476 (4)	
C25	−0.16522 (13)	−0.0609 (2)	0.20663 (8)	0.0592 (5)	
H25	−0.1784	−0.1142	0.2360	0.071*	
C26	−0.22817 (13)	0.0369 (2)	0.17457 (9)	0.0642 (5)	
H26	−0.2843	0.0499	0.1819	0.077*	
C27	−0.20917 (13)	0.1186 (2)	0.13057 (9)	0.0630 (5)	
H27	−0.2526	0.1860	0.1089	0.076*	
C28	−0.12729 (12)	0.1001 (2)	0.11913 (8)	0.0546 (4)	
H28	−0.1154	0.1559	0.0899	0.066*	
C29	−0.06034 (11)	−0.00243 (17)	0.15091 (7)	0.0442 (4)	
C30	0.04095 (12)	0.0481 (2)	0.08884 (8)	0.0521 (4)	
H30	−0.0033	0.0281	0.0531	0.063*	
C31	0.23628 (12)	0.27271 (18)	0.13895 (8)	0.0480 (4)	
C32	0.31301 (12)	0.29697 (18)	0.19424 (8)	0.0497 (4)	
C33	0.32713 (12)	0.22094 (19)	0.24690 (8)	0.0521 (4)	
C34	0.40499 (14)	0.2494 (2)	0.29426 (9)	0.0671 (5)	
H34	0.4144	0.1974	0.3289	0.080*	
C35	0.46828 (16)	0.3539 (3)	0.29042 (10)	0.0822 (7)	
H35	0.5207	0.3714	0.3223	0.099*	
C36	0.45451 (17)	0.4331 (3)	0.23953 (11)	0.0847 (7)	
H36	0.4967	0.5056	0.2373	0.102*	
C37	0.37807 (15)	0.4044 (2)	0.19212 (10)	0.0672 (5)	
H37	0.3694	0.4575	0.1578	0.081*	
C38	0.23682 (15)	−0.2434 (3)	0.19098 (11)	0.0880 (7)	
H38A	0.2578	−0.2118	0.2310	0.132*	
H38B	0.2881	−0.2436	0.1757	0.132*	
H38C	0.2116	−0.3413	0.1887	0.132*	
H2	0.2587 (14)	0.302 (2)	0.4481 (8)	0.080*	
H4A	0.1781 (14)	0.116 (2)	0.1714 (6)	0.080*	

### Atomic displacement parameters (Å^2^)

**Table:**

	*U*^11^	*U*^22^	*U*^33^	*U*^12^	*U*^13^	*U*^23^
O1	0.0480 (7)	0.0460 (7)	0.0824 (9)	−0.0025 (5)	0.0255 (6)	0.0106 (6)
O2	0.0597 (8)	0.0567 (7)	0.0590 (8)	−0.0150 (6)	0.0236 (6)	−0.0042 (6)
O3	0.0675 (8)	0.0794 (9)	0.0541 (8)	−0.0345 (7)	0.0264 (7)	−0.0193 (7)
O4	0.0550 (7)	0.0666 (8)	0.0763 (9)	0.0205 (6)	0.0238 (7)	0.0149 (7)
O5	0.0687 (8)	0.0665 (8)	0.0698 (9)	0.0089 (7)	0.0296 (7)	0.0261 (7)
O6	0.0772 (9)	0.0642 (8)	0.0499 (7)	−0.0102 (7)	0.0206 (7)	0.0054 (6)
N1	0.0416 (7)	0.0511 (8)	0.0539 (8)	−0.0098 (6)	0.0173 (6)	0.0013 (7)
N2	0.0391 (7)	0.0490 (8)	0.0520 (8)	−0.0114 (6)	0.0147 (6)	−0.0003 (6)
N3	0.0466 (8)	0.0639 (9)	0.0484 (8)	0.0113 (7)	0.0186 (7)	0.0105 (7)
N4	0.0476 (8)	0.0555 (9)	0.0464 (8)	0.0072 (7)	0.0184 (7)	0.0101 (7)
C1	0.0385 (8)	0.0446 (8)	0.0400 (8)	−0.0099 (7)	0.0111 (6)	−0.0020 (7)
C2	0.0409 (8)	0.0443 (9)	0.0486 (9)	−0.0065 (7)	0.0157 (7)	−0.0011 (7)
C3	0.0491 (10)	0.0491 (10)	0.0649 (11)	−0.0122 (8)	0.0236 (8)	0.0033 (8)
C4	0.0408 (9)	0.0646 (11)	0.0652 (12)	−0.0126 (8)	0.0220 (8)	0.0004 (9)
C5	0.0419 (9)	0.0572 (10)	0.0454 (9)	−0.0044 (8)	0.0133 (7)	−0.0042 (8)
C6	0.0471 (10)	0.0761 (13)	0.0607 (12)	0.0048 (9)	0.0171 (9)	−0.0025 (10)
C7	0.0623 (13)	0.0728 (14)	0.0696 (13)	0.0173 (11)	0.0147 (10)	0.0008 (11)
C8	0.0721 (14)	0.0506 (11)	0.0779 (14)	0.0060 (10)	0.0107 (11)	0.0091 (10)
C9	0.0511 (10)	0.0521 (10)	0.0656 (12)	−0.0050 (8)	0.0127 (9)	0.0063 (9)
C10	0.0418 (9)	0.0471 (9)	0.0402 (9)	−0.0058 (7)	0.0094 (7)	−0.0032 (7)
C11	0.0387 (8)	0.0466 (9)	0.0437 (9)	−0.0078 (7)	0.0114 (7)	0.0038 (7)
C12	0.0473 (9)	0.0400 (8)	0.0396 (9)	−0.0079 (7)	0.0079 (7)	0.0058 (7)
C13	0.0431 (8)	0.0412 (8)	0.0379 (8)	−0.0087 (7)	0.0068 (6)	0.0089 (7)
C14	0.0487 (9)	0.0484 (9)	0.0398 (9)	−0.0128 (7)	0.0079 (7)	0.0032 (7)
C15	0.0545 (10)	0.0672 (12)	0.0537 (11)	−0.0150 (9)	0.0216 (8)	0.0020 (9)
C16	0.0521 (11)	0.0772 (13)	0.0647 (12)	−0.0234 (10)	0.0198 (9)	0.0046 (10)
C17	0.0616 (12)	0.0699 (12)	0.0619 (12)	−0.0324 (10)	0.0155 (10)	−0.0068 (10)
C18	0.0577 (11)	0.0516 (10)	0.0491 (10)	−0.0160 (8)	0.0138 (8)	−0.0034 (8)
C19	0.0717 (14)	0.0544 (12)	0.123 (2)	0.0039 (10)	0.0378 (13)	0.0286 (12)
C20	0.0449 (9)	0.0469 (9)	0.0435 (9)	−0.0001 (7)	0.0118 (7)	−0.0018 (7)
C21	0.0484 (9)	0.0473 (9)	0.0519 (10)	0.0037 (8)	0.0139 (8)	−0.0013 (8)
C22	0.0642 (12)	0.0476 (10)	0.0541 (11)	0.0044 (8)	0.0102 (9)	0.0064 (8)
C23	0.0714 (12)	0.0505 (10)	0.0500 (10)	−0.0098 (9)	0.0212 (9)	0.0014 (8)
C24	0.0513 (10)	0.0445 (9)	0.0467 (9)	−0.0120 (7)	0.0150 (8)	−0.0108 (7)
C25	0.0604 (11)	0.0633 (11)	0.0596 (11)	−0.0223 (10)	0.0270 (9)	−0.0171 (9)
C26	0.0452 (10)	0.0747 (13)	0.0742 (13)	−0.0112 (10)	0.0211 (10)	−0.0234 (11)
C27	0.0462 (10)	0.0645 (12)	0.0744 (13)	0.0003 (9)	0.0133 (9)	−0.0095 (10)
C28	0.0449 (10)	0.0568 (10)	0.0591 (11)	−0.0014 (8)	0.0117 (8)	−0.0015 (9)
C29	0.0429 (9)	0.0427 (8)	0.0453 (9)	−0.0057 (7)	0.0113 (7)	−0.0083 (7)
C30	0.0458 (9)	0.0649 (11)	0.0446 (9)	0.0091 (9)	0.0126 (7)	0.0049 (8)
C31	0.0503 (10)	0.0434 (9)	0.0595 (11)	0.0140 (8)	0.0307 (8)	0.0099 (8)
C32	0.0547 (10)	0.0458 (9)	0.0576 (10)	0.0058 (8)	0.0304 (8)	−0.0035 (8)
C33	0.0587 (11)	0.0502 (9)	0.0544 (11)	0.0022 (8)	0.0281 (9)	−0.0053 (8)
C34	0.0711 (13)	0.0813 (14)	0.0524 (11)	−0.0015 (11)	0.0246 (10)	−0.0123 (10)
C35	0.0760 (15)	0.1089 (19)	0.0671 (14)	−0.0206 (14)	0.0304 (12)	−0.0308 (13)
C36	0.0874 (16)	0.0939 (17)	0.0848 (17)	−0.0348 (13)	0.0443 (14)	−0.0277 (14)
C37	0.0785 (14)	0.0622 (12)	0.0746 (14)	−0.0090 (10)	0.0436 (12)	−0.0052 (10)
C38	0.0680 (14)	0.0909 (16)	0.1037 (19)	0.0353 (12)	0.0250 (13)	0.0250 (14)

### Geometric parameters (Å, °)

**Table:**

O1—C2	1.3631 (19)	C15—H15	0.9300
O1—C19	1.419 (2)	C16—C17	1.375 (3)
O2—C12	1.2401 (19)	C16—H16	0.9300
O3—C14	1.3635 (19)	C17—C18	1.378 (2)
O3—H3	0.8200	C17—H17	0.9300
O4—C21	1.370 (2)	C18—H18	0.9300
O4—C38	1.426 (2)	C19—H19A	0.9600
O5—C31	1.2208 (19)	C19—H19B	0.9600
O6—C33	1.365 (2)	C19—H19C	0.9600
O6—H6	0.8200	C20—C21	1.375 (2)
N1—C11	1.283 (2)	C20—C29	1.426 (2)
N1—N2	1.3801 (18)	C20—C30	1.477 (2)
N2—C12	1.348 (2)	C21—C22	1.407 (2)
N2—H2	0.918 (19)	C22—C23	1.357 (3)
N3—C30	1.275 (2)	C22—H22	0.9300
N3—N4	1.376 (2)	C23—C24	1.405 (2)
N4—C31	1.360 (2)	C23—H23	0.9300
N4—H4A	0.895 (9)	C24—C25	1.418 (2)
C1—C2	1.389 (2)	C24—C29	1.419 (2)
C1—C10	1.426 (2)	C25—C26	1.354 (3)
C1—C11	1.469 (2)	C25—H25	0.9300
C2—C3	1.407 (2)	C26—C27	1.397 (3)
C3—C4	1.357 (2)	C26—H26	0.9300
C3—H3A	0.9300	C27—C28	1.366 (2)
C4—C5	1.406 (2)	C27—H27	0.9300
C4—H4	0.9300	C28—C29	1.412 (2)
C5—C6	1.417 (2)	C28—H28	0.9300
C5—C10	1.425 (2)	C30—H30	0.9300
C6—C7	1.351 (3)	C31—C32	1.486 (3)
C6—H6A	0.9300	C32—C37	1.399 (3)
C7—C8	1.396 (3)	C32—C33	1.401 (2)
C7—H7	0.9300	C33—C34	1.386 (3)
C8—C9	1.361 (3)	C34—C35	1.373 (3)
C8—H8	0.9300	C34—H34	0.9300
C9—C10	1.419 (2)	C35—C36	1.381 (3)
C9—H9	0.9300	C35—H35	0.9300
C11—H11	0.9300	C36—C37	1.374 (3)
C12—C13	1.489 (2)	C36—H36	0.9300
C13—C18	1.393 (2)	C37—H37	0.9300
C13—C14	1.403 (2)	C38—H38A	0.9600
C14—C15	1.385 (2)	C38—H38B	0.9600
C15—C16	1.365 (3)	C38—H38C	0.9600
			
C2—O1—C19	119.52 (13)	O1—C19—H19A	109.5
C14—O3—H3	109.5	O1—C19—H19B	109.5
C21—O4—C38	119.16 (15)	H19A—C19—H19B	109.5
C33—O6—H6	109.5	O1—C19—H19C	109.5
C11—N1—N2	114.42 (14)	H19A—C19—H19C	109.5
C12—N2—N1	118.72 (14)	H19B—C19—H19C	109.5
C12—N2—H2	121.3 (13)	C21—C20—C29	119.43 (15)
N1—N2—H2	120.0 (13)	C21—C20—C30	121.17 (15)
C30—N3—N4	116.65 (14)	C29—C20—C30	119.14 (14)
C31—N4—N3	117.96 (14)	O4—C21—C20	115.65 (15)
C31—N4—H4A	119.5 (14)	O4—C21—C22	123.30 (15)
N3—N4—H4A	122.0 (14)	C20—C21—C22	121.03 (16)
C2—C1—C10	119.50 (14)	C23—C22—C21	119.64 (17)
C2—C1—C11	116.74 (14)	C23—C22—H22	120.2
C10—C1—C11	123.76 (14)	C21—C22—H22	120.2
O1—C2—C1	116.21 (13)	C22—C23—C24	122.10 (16)
O1—C2—C3	122.76 (14)	C22—C23—H23	119.0
C1—C2—C3	121.02 (15)	C24—C23—H23	119.0
C4—C3—C2	119.53 (16)	C23—C24—C25	122.61 (17)
C4—C3—H3A	120.2	C23—C24—C29	118.35 (15)
C2—C3—H3A	120.2	C25—C24—C29	119.03 (16)
C3—C4—C5	122.26 (15)	C26—C25—C24	120.98 (18)
C3—C4—H4	118.9	C26—C25—H25	119.5
C5—C4—H4	118.9	C24—C25—H25	119.5
C4—C5—C6	121.23 (16)	C25—C26—C27	120.25 (18)
C4—C5—C10	118.70 (15)	C25—C26—H26	119.9
C6—C5—C10	120.07 (16)	C27—C26—H26	119.9
C7—C6—C5	121.20 (18)	C28—C27—C26	120.50 (19)
C7—C6—H6A	119.4	C28—C27—H27	119.8
C5—C6—H6A	119.4	C26—C27—H27	119.8
C6—C7—C8	119.15 (18)	C27—C28—C29	121.10 (18)
C6—C7—H7	120.4	C27—C28—H28	119.4
C8—C7—H7	120.4	C29—C28—H28	119.4
C9—C8—C7	121.74 (19)	C28—C29—C24	118.11 (15)
C9—C8—H8	119.1	C28—C29—C20	122.44 (15)
C7—C8—H8	119.1	C24—C29—C20	119.44 (15)
C8—C9—C10	121.28 (17)	N3—C30—C20	129.77 (16)
C8—C9—H9	119.4	N3—C30—H30	115.1
C10—C9—H9	119.4	C20—C30—H30	115.1
C9—C10—C5	116.55 (15)	O5—C31—N4	120.90 (17)
C9—C10—C1	124.44 (15)	O5—C31—C32	121.51 (16)
C5—C10—C1	118.99 (14)	N4—C31—C32	117.56 (15)
N1—C11—C1	121.33 (15)	C37—C32—C33	117.81 (18)
N1—C11—H11	119.3	C37—C32—C31	115.67 (17)
C1—C11—H11	119.3	C33—C32—C31	126.49 (16)
O2—C12—N2	121.91 (15)	O6—C33—C34	120.55 (17)
O2—C12—C13	121.29 (14)	O6—C33—C32	119.19 (16)
N2—C12—C13	116.80 (15)	C34—C33—C32	120.26 (17)
C18—C13—C14	117.65 (15)	C35—C34—C33	120.4 (2)
C18—C13—C12	116.01 (15)	C35—C34—H34	119.8
C14—C13—C12	126.31 (14)	C33—C34—H34	119.8
O3—C14—C15	120.06 (15)	C34—C35—C36	120.3 (2)
O3—C14—C13	119.73 (14)	C34—C35—H35	119.8
C15—C14—C13	120.20 (15)	C36—C35—H35	119.8
C16—C15—C14	120.86 (18)	C37—C36—C35	119.6 (2)
C16—C15—H15	119.6	C37—C36—H36	120.2
C14—C15—H15	119.6	C35—C36—H36	120.2
C15—C16—C17	119.94 (17)	C36—C37—C32	121.5 (2)
C15—C16—H16	120.0	C36—C37—H37	119.2
C17—C16—H16	120.0	C32—C37—H37	119.2
C16—C17—C18	120.04 (17)	O4—C38—H38A	109.5
C16—C17—H17	120.0	O4—C38—H38B	109.5
C18—C17—H17	120.0	H38A—C38—H38B	109.5
C17—C18—C13	121.31 (17)	O4—C38—H38C	109.5
C17—C18—H18	119.3	H38A—C38—H38C	109.5
C13—C18—H18	119.3	H38B—C38—H38C	109.5

### Hydrogen-bond geometry (Å, °)

**Table:**

*D*—H···*A*	*D*—H	H···*A*	*D*···*A*	*D*—H···*A*
O3—H3···N3^i^	0.82	2.24	3.0049 (18)	155
O3—H3···O5^i^	0.82	2.30	2.914 (2)	133
O6—H6···O2	0.82	1.87	2.6683 (17)	164
N2—H2···O3	0.918 (19)	1.942 (16)	2.6518 (18)	132.7 (17)
N4—H4A···O6	0.895 (9)	1.948 (16)	2.6581 (19)	135.0 (18)
N4—H4A···O4	0.895 (9)	2.38 (2)	2.877 (2)	114.9 (16)

Symmetry codes: (i) *x*, −*y*+1/2, *z*+1/2.
